# Disease Network-Based Approaches to Study Comorbidity in Heart Failure: Current State and Future Perspectives

**DOI:** 10.1007/s11897-024-00693-7

**Published:** 2024-12-27

**Authors:** Sergio Alejandro Gomez-Ochoa, Jan D. Lanzer, Rebecca T. Levinson

**Affiliations:** 1 Department of General Internal Medicine and Psychosomatics, Heidelberg University Hospital, Im Neuenheimer Feld 410, 69120 Heidelberg, Germany; 2 Institute for Computational Biomedicine, Faculty of Medicine, Heidelberg University, Heidelberg University Hospital, Heidelberg, Germany

**Keywords:** Networks, Comorbidity, Heart failure

## Abstract

**Purpose of Review:**

Heart failure (HF) is often accompanied by a constellation of comorbidities, leading to diverse patient presentations and clinical trajectories. While traditional methods have provided valuable insights into our understanding of HF, network medicine approaches seek to leverage these complex relationships by analyzing disease at a systems level. This review introduces the concepts of network medicine and explores the use of comorbidity networks to study HF and heart disease.

**Recent Findings:**

Comorbidity networks are used to understand disease trajectories, predict outcomes, and uncover potential molecular mechanisms through identification of genes and pathways relevant to comorbidity. These networks have shown the importance of non-cardiovascular comorbidities to the clinical journey of patients with HF. However, the community should be aware of important limitations in developing and implementing these methods.

**Summary:**

Network approaches hold promise for unraveling the impact of comorbidities in the complex presentation and genetics of HF. Methods that consider comorbidity presence and timing have the potential to help optimize management strategies and identify pathophysiological mechanisms.

## Introduction

The emergence of big data in the cardiology field has presented an unprecedented opportunity to unravel the complexities underlying cardiovascular diseases [1]. Heart failure (HF), characterized by its high prevalence, heterogeneous nature, and diverse clinical presentations, represents an ideal candidate for the implementation of new big data-derived approaches [2–4]. While traditional reductionist methods have provided valuable insights into our understanding of the disease so far, they often struggle to fully capture the systemic nature of HF and its associated comorbidities [5]. Network medicine offers a complementary analytical framework that aims to leverage this inherent heterogeneity [6]. By analyzing relationships between multiple diseases/conditions simultaneously, these methods can reveal patterns that may not be immediately apparent in conventional epidemiological studies [7]. This network-based view aligns with the growing understanding of HF as a systemic disorder rather than a purely cardiac condition [8, 9]. In this review, we examine the current state of disease and comorbidity networks studying HF, discussing the methodological foundations, key findings, and potential clinical implications of the literature. We also critically assess the limitations of these approaches and consider future directions that may enhance their utility in research and clinical practice.

### Comorbidities in Heart Failure

Comorbidity is the presence of additional complicating disorders in patients with a primary disease of interest [10]. In heart failure (HF) and its subphenotypes, comorbidity is common in patient populations across the world [11]. In some studies, more than 85% of patients reported having at least two conditions in addition to HF [12, 13]. Despite this, there is no agreed-upon definition of comorbidity in terms of causal relationship to the primary disease of interest [14], and studies use different definitions, which place varying degrees of importance on the index disease [15], causality, and presence of additional conditions [16]. When multiple diseases are present in addition to the condition of interest, the term multimorbidity is frequently used [17].

Patients with HF can experience a wide variety of comorbid conditions. However, most investigations have focused on a small number of the most common comorbidities. Commonly assessed comorbidities include coronary artery disease, hypertension, diabetes, atrial fibrillation, chronic obstructive pulmonary disorder, kidney disease, and obesity, amongst others [18–22]. In patients with HF, comorbidity is associated with poorer quality of life [23], increased complications, and higher rates of readmission and death [18, 24–26]. Noncardiovascular comorbidities especially have been more strongly associated with risk of death and hospitalization [27, 28].

Comorbidities have been particularly important in the context of subtypes of HF.Patients with HFare commonly grouped by left ventricular ejection fraction, with patients with an LVEF ≤ 40 identified as HF with reduced ejection fraction (HFrEF) and those with an LVEF ≥ 50 categorized as HF with preserved ejection fraction (HFpEF) [29]. The differences in comorbidity in these two groups have been posited as impacting changes in the heart before disease [30], and guidelines for managing specific comorbidities, such as hypertension or diabetes, play a particularly crucial role in tailoring treatment strategies for patients with HFpEF, as these comorbidities often contribute significantly to the pathophysiology and symptom burden in this subtype of heart failure [31, 32]. Some literature has found that HFpEF patients have higher rates of comorbidity [28], and especially non-cardiac comorbidities, than their HFrEF counterparts [33–35], while others have found prevalence rates across EF-based subtypes to be fairly consistent [36]. Comorbidities common in HFrEF have been described as more likely to be cardiac diseases [37] than those in HFpEF patients. There is also some evidence that the relationship between comorbidity and negative outcomes such as mortality [38] differs between EF-based subtypes [36, 39, 40]. The relationship between comorbidities and HF subtype can be complicated by age and sex [41]. For example, anemia is a more frequent comorbidity in HFpEF compared to HFrEF, and is more common in women with HF regardless of subtype than men [42].

### Leveraging Network Medicine to Understanding Comorbidities in Disease

The desire to understand the complex relationships between comorbidity and HF, and the availability of big data resources [43] has resulted in embracing methods that can leverage this information. Network medicine can be defined as the application of network methodologies to approach the study of human diseases [6]. First applied to study physical interactions at the molecular level within the cell [44, 45], investigators soon adapted these methods to explore human disease relationships by hypothesizing that diseases sharing molecular characteristics might also display phenotypic similarities [46]. Network medicine relies on the hypothesis that if two diseases are related, changes in the network that cause one disease will likely affect the manifestation of other diseases as well [47].

**Fig. 1:**
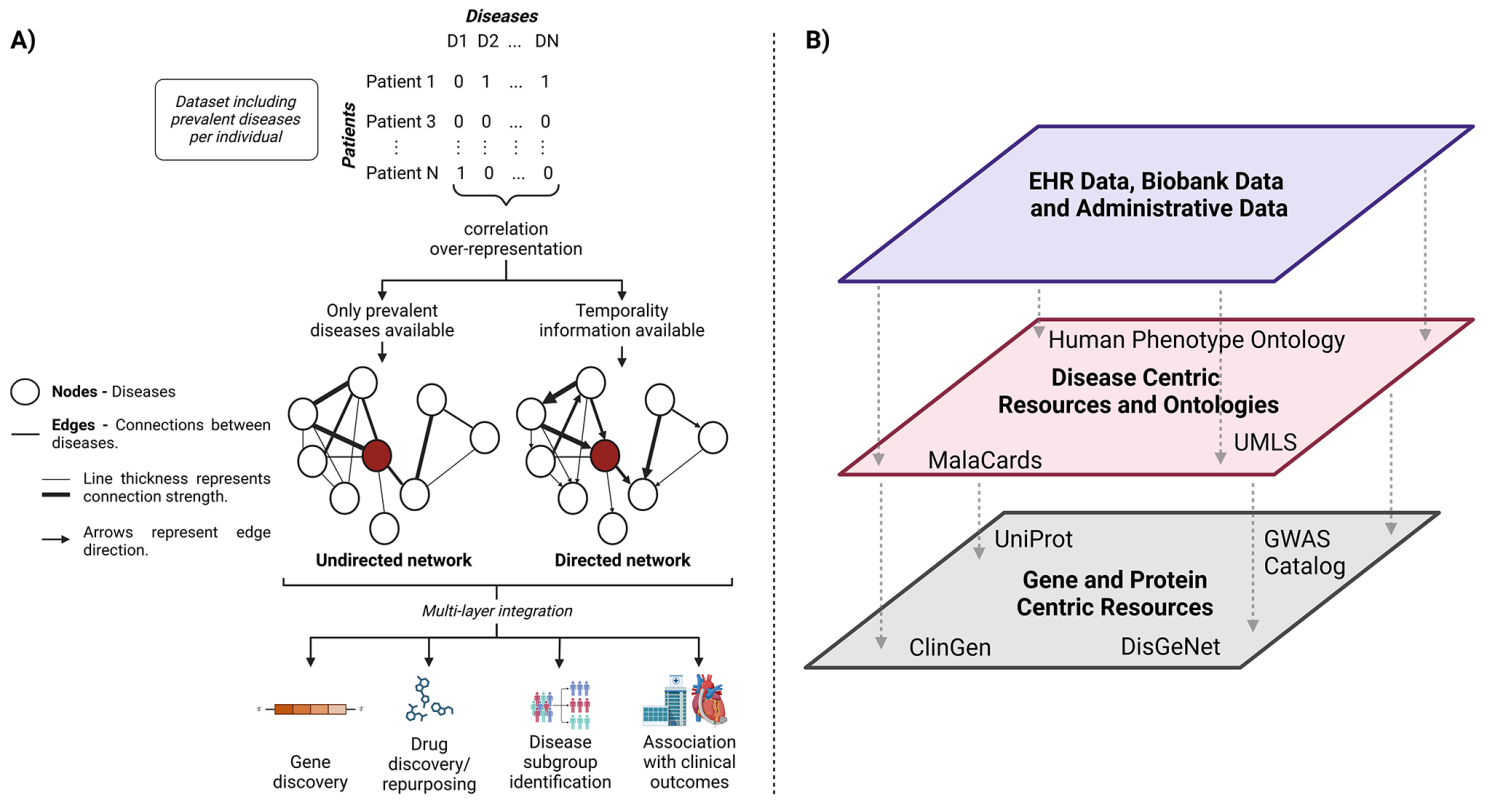
Overview of methods of network construction. Networks can be constructed (**A**) from data where information on each disease is present for each patient. In these networks, diseases are represented by nodes and the connections between diseases by edges. After network construction they can be used for a variety of study types or can be (**B**) integrated with data or prior knowledge resources that allow the linking of different levels of information about medicine and biology

Conceptually, a network is a collection of nodes connected by edges [47]. Networks can be undirected, directed, and/or weighted, with edges providing information about the directionality and strength of the relationship between the nodes [48] (Fig. 1A). In the case of comorbidity networks, the network nodes represent diseases and the edges depict connections between two nodes, often representing a correlation between or an observed-to-expected ratio of two diseases [46, 49, 50]. Several types of measures including degree characterize networks, measuring the number of connections in a node, betweenness, a measure of the number of nodes a node can influence, closeness, measuring the average from a node to the rest of the nodes in the network [48]. A list of network-related vocabulary can be found in the included Glossary of Terms.

To construct a comorbidity network, the definition of nodes and edges must align with the research question. In comorbidity networks, the nodes are typically based on a disease ontology. Since many ontologies exist (e.g. MESH, HPO, ICD, PheWAS, DO), each developed for different purposes and with different levels of resolution, selecting the appropriate one requires careful consideration of the research question. Sensitivity analyses have shown that ontology choice significantly influences network topology [51]. Then, statistical tests are often applied to assess whether two diseases co-occur more frequently than expected based on their prevalence [49]. These models can be enhanced in various ways including incorporating temporal relationships [52, 53] or multivariate analyses to control for confounders [54, 55]. From these models, edges are filtered, usually by selecting cutoffs based on effect sizes or p-values globally (on network level) or locally (e.g. on node level [56]). In traditional comorbidity networks, diseases serve as the nodes, and statistical associations between them form the edges. However, alternative approaches, such as patient networks, can be used for patient stratification or classification, where the nodes represent patients and the edges reflect the similarity [57, 58]of their comorbidity profiles [59]. A central challenge in both approaches is designing a network that accurately captures the relationships under study, particularly in defining meaningful edges that reflect comorbid associations.

Once built, networks can be layered with other types of network data to create “multi-layer” networks [60](Fig. 1B). These can be homogeneous if only one node type is used, or heterogeneous when different types of networks are combined. Heterogeneous multi-layer networks have been suggested to integrate clinical information, omics, and more to identify genes and proteins involved in pathological processes [57, 58]. Prior knowledge is one important source of information that links phenotypic data to biological knowledge [61]. Resources such as DISGENET [62], Malacards [63], UniProtKB [64], and ClinVar [65], amongst others, can provide important information about linking phenotypic information to disease level knowledge, and disease ontologies [66, 67] and language systems [68] can help combine knowledge from different data sources with different nomenclatures.

Comorbidity networks often use large numbers of comorbidities, and therefore, most analyses are performed in datasets from electronic health records or administrative data. These data sources allow the capture of a broad spectrum of comorbid conditions in populations who have actively sought care. However, some network analyses only analyze a small spectrum of comorbidity, creating a phenotype algorithm for each one [69, 70]. At the core, though, these methods focus on the interconnectedness of comorbidities, aiming to provide insights into the complex web of comorbid relationships and potential pathways between different diseases [6, 71] rather than make patient-level statements.

Other common computational methods in the study of the heterogeneity of HF, such as clustering [72, 73], latent class analyses [74, 75], and factor analyses [13], have been used as alternatives to, or in combination with, comorbidity network analysis as they can provide easily interpretable insights about patient sub-populations. These methods focus on patients to group individuals based on their shared characteristics without necessarily exploring the intricate relationships between the variables themselves [76]. We will only loosely touch on these methods as they intersect with using networks to study HF comorbidities. We recommend these reviews for readers interested in the methodology behind clustering in HF [77–80].

### Comorbidity Networks of Heart Failure and Heart Disease

Networks have been used to investigate different aspects of HF. In the following sections, we discuss networks developed in HF and one of its most frequent underlying etiologies, ischemic heart disease (IHD). We have loosely grouped literature by the underlying purpose for which the network was developed.

#### Networks have Evaluated Comorbidity Relationships in HF and IHD

An important use of disease networks is to explore the connections between comorbidities in disease subpopulations. *Carmona-Perez et al.* evaluated the comorbidity patterns in HF and COPD patients, specifically comparing men and women [81] with one or both diseases. Some diseases, such as kidney disease and respiratory disorders, were amongst the 10 most highly connected nodes in the HF network of both males and females. Others were more sex specific, with arthritis one of the 10 nodes with the highest links in the HF network in women only and peripheral vascular disorder only in the male-only HF network.

Networks developed within age and sex-based subgroups have shown that comorbidity relationships may be different in independent populations. In an IHD network, males had a more complex network than females, and the connections between comorbidities were also different [82]. For example, conditions relating to cerebrovascular disease were more common in men, while kidney failure and related alterations of metabolism were more comorbid in females. Across age-stratified networks, they found overall consistency of prevalent comorbidities, but that comorbidity relationships changed, becoming more complicated in older patients. A study by *Martins et al.* used clustering as the primary method to determine groups of associated comorbidity, but leveraged network methods to visualize the results of these clusters [83]. The network visualization revealed differences in comorbidity prevalence and complexity between clusters and allowed important disease pairs in the clusters to be identified. The relationship between anemia and hypertension and anemia and CKD was important in one cluster, while in another the connections of AF, to obesity and hypertension were important.

This was also evident in a study from the Colombian heart failure registry [84]. In this study, sex- and LVEF-stratified networks revealed significant differences in the relationships between comorbidities, highlighting significant correlations between valvular disease and atrial fibrillation, as well as valvular disease and coronary heart disease exclusive to women, while the correlation between valvular disease and T2DM was exclusive to men. Moreover, a significant correlation between chronic kidney disease and valvular disease was observed only in HFrEF patients, while the strength of the correlation between T2DM and chronic kidney disease in HFpEF almost doubled the one in HFrEF.

Finally, at least one example has shown that in populations of patients with HF grouped over chronological time, the relationship between comorbidities in HF has changed over time, but the disease communities have remained stable [85].

#### Communities in Comorbidity Networks have been Associated with Outcomes

Comorbidity networks have also been leveraged to provide information about the risk of clinically relevant outcomes. A large study of the Swedish Heart Failure Registry used graphical models to assess the relationship between comorbidities and metrics of patient health including patient-reported outcomes. It found non-cardiovascular comorbidities were a major driver of patient health and that for some patients might be driving the burden of symptoms normally assessed in HF [86]. Networks have also been used to study the usage of clinical services by patients with HF, demonstrating a diffuse flow of patients between clinical resources, but one that changed depending on whether a patient had been admitted [87].

Regarding “hard” clinical outcomes, *Zheng et al.* used latent class analysis to subgroup patients with HFpEF and HFrEF by comorbidities. Then, they used network methods to examine the relationships between the comorbid conditions in each cluster. They found that different connections between common comorbidities were important in each cluster, but the relevant comorbidities were highly overlapping. Comorbidity-based populations were associated with differential outcomes rates, including all-cause mortality and readmission, despite otherwise similar clinical characteristics. They used this to argue for hierarchical comorbidity management in chronic heart failure patients [88].

Similarly, a study in IHD used network analyses and machine learning to predict which disease clusters would put patients at risk of progression to heart failure. They built a disease co-occurrence network for IHD and personal networks for individual patients. They used network measures as features in machine learning models, concluding that network measures were more important as features than demographic information such as age and sex [89]. Finally, at least one study used networks to identify diagnoses and procedures associated with increased hospital costs for affected patients [90].

#### Networks to Connect HF to Genes

While the number of studies that have analyzed ejection fraction-based subtypes of HF using clustering methods is quite large, few studies were primarily network-based. In our previous work, we built a comorbidity network from patients with HF visiting a German university hospital [91]. Ejection fraction data was then used to subphenotype HFpEF and HFrEF patients and learn discriminant comorbidity profiles. Statistical methods were employed to contrast disease communities more representative of the HFpEF and HFrEF populations. We found that the characteristics of HFpEF patients were overrepresented in the endocrine and pulmonary disease clusters. Moreover, using existing data about the relationship of diseases to genes, we developed a heterogeneous multi-layer network and used a random walk to predict which genes were closer in the network context to the comorbidity profiles discriminant for HFpEF or HFrEF. As a result, we identified genes involved in fibrosis, hypertrophy, oxidative stress, and endoplasmic reticulum stress, which were significantly overrepresented in a murine transcriptomic disease signature of HFpEF, providing additional support for their relevance in this context. This work serves as a proof of concept, suggesting that not only can comorbid diseases share molecular profiles, but multi-organ syndromes like HFpEF may be linked to recurrent molecular patterns across organs. However, studying systemic diseases remains challenging, and experimental strategies are particularly needed to evaluate organism-wide changes in response to disease.

On the other hand, *Cruz-Ávila et al.* built comorbidity networks for a CVD population and divided the population into age brackets as determined by 10-year increments [92]. In their network, they found that arrhythmias, heart failure, and kidney disease had the greatest number of connections. They noted that many possible links between comorbidities were absent and that comorbidity relationships were heterogeneous. Additionally, they reported that congenital disorders had a prominent place in the network in children, while in adults, complex diseases became the central nodes. To identify comorbidity-associated genes, they used ClinVar to link diseases to genetics and then performed a pathway analysis. HF was well connected in age-stratified networks from age 11 on.

#### Heart Failure in Networks Focused on Other Diseases

Although not designed to assess HF specifically, networks focused on other diseases or generally on multimorbid patients without a specific index disease have also captured HF and its subtypes. This may be important for highlighting comorbidities before HF [93] and understanding HF in patients with complex healthcare trajectories. Querying these networks may provide valuable insights for identifying at-risk patients or interventional opportunities before the onset of heart failure.

Morbinet, while focused on type 2 diabetes, captured heart failure, heart disease, and ischemic heart disease as important nodes [94]. Heart failure was most strongly linked with diabetes and pulmonary heart disease, though it was also less strongly linked to non-cardiovascular comorbidities, including gout and liver disease. A second network built for type 2 diabetes also identified HF as an important node in a cardiovascular disease-heavy cluster and found that diseases in the cluster were less common in the networks of non-diabetics [95]. This cluster contained comorbidities such as anemia, lymphoma, and rheumatoid arthritis in addition to circulatory and vascular disorders. These networks demonstrate the complicated relationship between diabetes and HF and the breadth of comorbidities associated with both. In contrast to the importance of HF in diabetes networks, in a study of lung cancer patients, the authors found that while the patients with HF all had multiple other comorbidities, HF itself did not play a strong role in the development of the comorbidity pattern an individual had [96]. This demonstrates that networks of other HF-relevant phenotypes will also recapitulate knowledge relevant to HF. Similarly, a network focused on hypothyroidism identified HF as a well-connected node that directionally led to hypothyroidism in the directed network [97].

General multimorbidity networks have also assessed mortality and hospitalization due to HF. A multimorbidity network in Veterans found differential rates of deceased persons 8 years after HF in different subclusters of the HF temporal network [98]. This demonstrates that comorbidity networks may be a valid way to identify high-risk populations. Other studies found differences in the number and length of stay in patients who develop HF depending on the order of CVD development [99]. However, another temporal network study found that hypertensive heart disease and heart failure were important nodes for predicting future heart failure, a result potentially at odds with some known developmental pathways for HF [100]. Data assessing multimorbidity in multiple racial/ethnic populations from the UK found that HF was associated with high multimorbidity coefficients in all populations [101]. They also found that many of the most common comorbidities in HF populations were exclusion criteria for clinical trials, providing an important assessment of the real-world applicability of trial data.

#### Limitations of Network Analyses

**Fig. 2:**
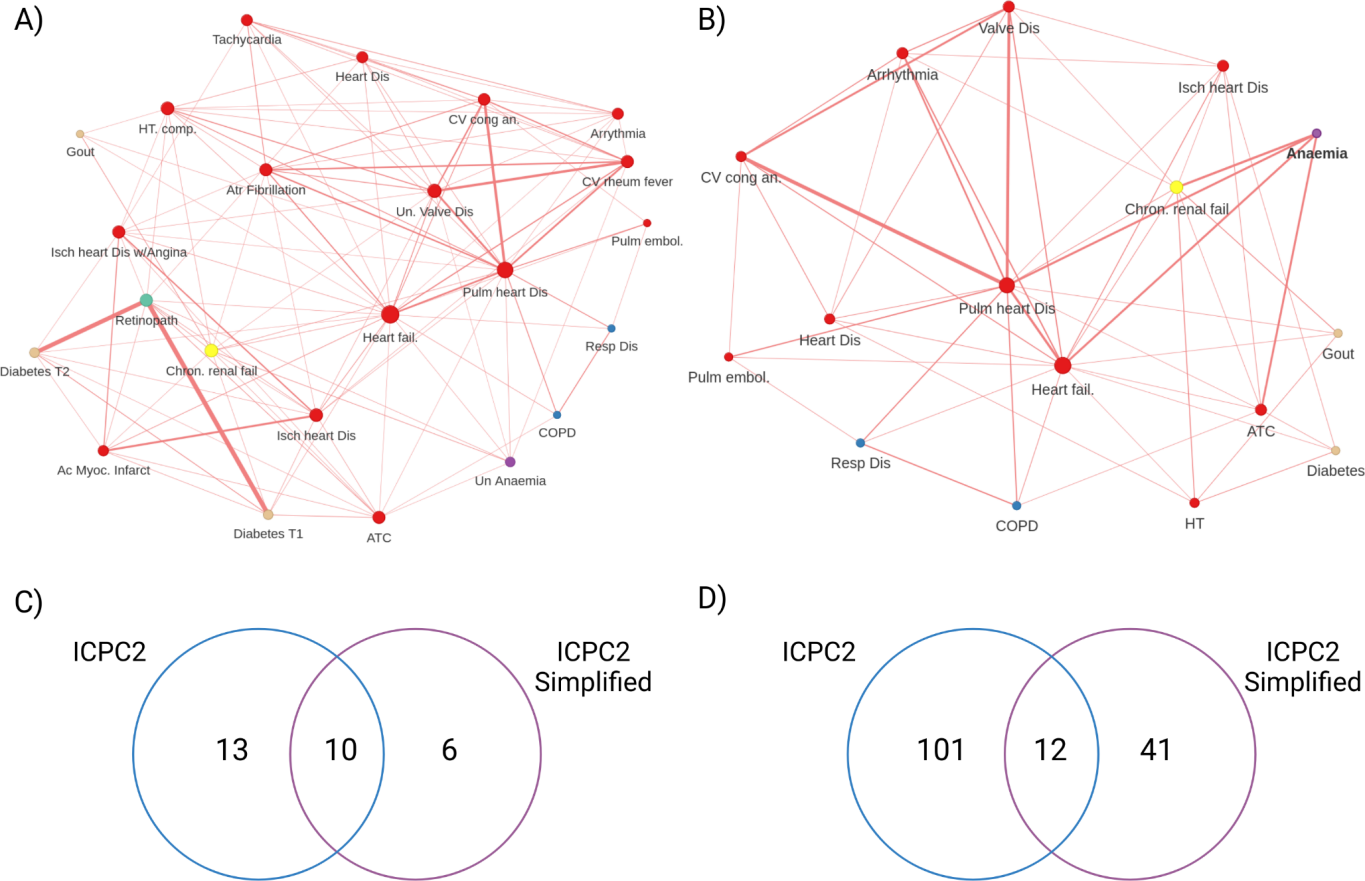
Comparison of two networks built using the Morbinet shiny browser [104] demonstrates how small processing changes can affect networks. The network built using an odds ratio for association of at least 1.8 with (**A**) International Classification of Primary Care, 2nd edition (ICPC2) codes contains more nodes and connections than that built with (**B**) simplified ICPC2 codes. Comparison of (**C**) number of shared nodes and (**D**) edges are shown in Venn Diagrams with ICPC2 data in blue and simplified ICPC2 data in purple

Network analyses can offer valuable insights into the complex landscape of comorbidities in HF; however, they also suffer from important limitations that researchers and clinicians alike must consider [6]. At their core, these approaches focus on disease-level relationships rather than individual patient characteristics, which can limit direct clinical applicability [102]. Moreover, the heavy reliance on diagnostic codes from electronic health records or administrative databases introduces potential biases and may not capture the full clinical picture, particularly for rare or underdiagnosed conditions [103]. The type of code and data preprocessing may also impact the network results (Fig. 2).

A significant challenge lies in the temporal and causal aspects of disease relationships. Many network analyses provide a static view, failing to account for the order of disease onset or the causal links between conditions. This limitation can hinder our understanding of disease progression and the development of targeted interventions [105]. Furthermore, the generalizability of these networks to diverse populations or healthcare settings may be limited, as they are often built using data from specific systems or regions [6].

Notably, the methodological aspects of network analyses also present challenges. The structure and insights derived from these networks can be highly sensitive to the specific metrics and algorithms used in their creation [102]. An example of this is provided in Fig. 2. This sensitivity makes comparisons across studies difficult and raises questions about the reproducibility of findings [105]. Unfortunately, external validation of network-derived insights in independent cohorts or through prospective studies is often lacking, limiting confidence in the generalizability of findings. Additionally, the focus on pairwise disease relationships in many analyses may oversimplify the complex interactions between multiple conditions [51], potentially missing important higher-order relationships [106].

Interpreting the clinical relevance of identified relationships remains a critical challenge [107]. Not all statistically significant connections in a network may be meaningful in a clinical context, and distinguishing between spurious associations and truly important relationships requires careful interpretation and validation. As networks become more complex, incorporating more diseases and relationships, they can become increasingly difficult to interpret and visualize effectively, potentially obscuring key insights [105, 108].

Finally, while theoretically possible, integrating diverse data types remains practically challenging. Combining clinical, genetic, and molecular data in a meaningful way is an ongoing area of research that has yet to be fully realized in many network studies [109]. This is compounded by a lack of consensus about terminology, which may limit the ability to incorporate datasets and compare results [110].

## Conclusion

**Fig. 3:**
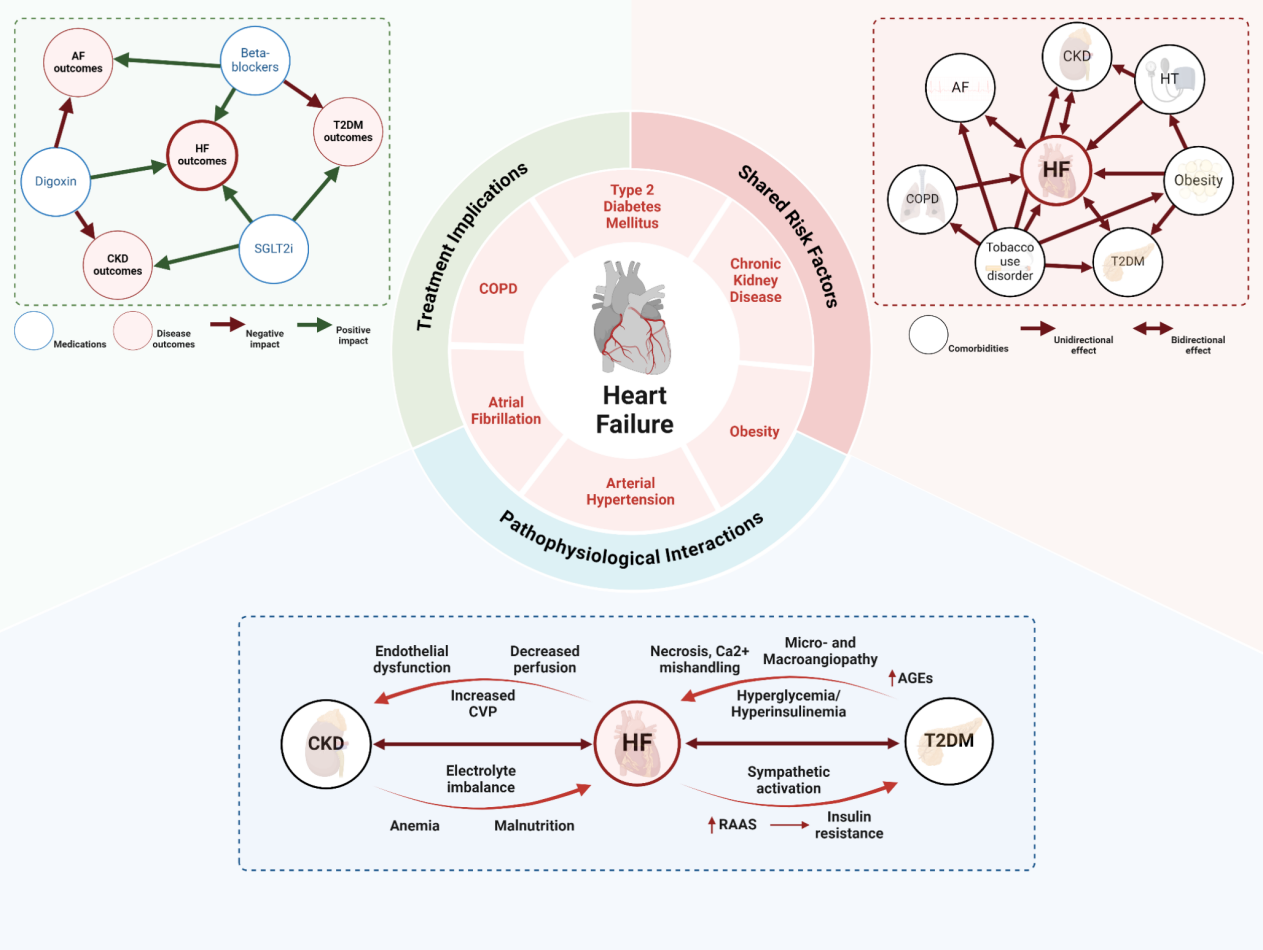
Schematic overview of potential of comorbidity network representations to explore the complexity and implications of comorbidities in heart failure. The central panel highlights the complex interplay between heart failure and its most common comorbidities, beyond the mere coexistence of diseases to clinical impact. The upper right panel highlights the commonality of risk associations derived from epidemiological studies between the different conditions surrounding HF. The network in the upper left panel represents the complex interplay between therapeutic classes and different outcomes across HF and related comorbidities, highlighting the differential impact of each therapy concerning the assessed outcome. Finally, the lower panel summarizes the pathophysiological interplay between HF, chronic kidney disease (CKD), and type 2 diabetes mellitus from a molecular perspective, highlighting bidirectional effects related to multisystemic pathways

Comorbidity networks can be leveraged to elucidate relationships between diseases that might otherwise go unrecognized. By harnessing disease heterogeneity at the individual level [58], network approaches have been important for advancing our understanding of the intricate connections between related diseases [111]. To fully exploit the potential of comorbidity networks, we must move beyond merely describing subpopulations and strive to make and validate claims about the epidemiology, pathophysiology, and potential avenues for treatment (Fig. 3). Linking networks to genomic data sources will be critical to understanding how the genetic heterogeneity in HF populations contributes to the variety of clinical trajectories patients experience and potentially identify treatment opportunities. This has been demonstrated even in patient populations with heart diseases that are often genetic but lack a single genetic driver [112]. Networks that delve into the biological underpinnings of disease subpopulations could pave the way for future innovations.

Disease networks also represent interactions with healthcare systems by representing comorbidity from clinical databases. Networks that consider the order and timing of diseases can provide information about clinical trajectories in patients with HF, even well before it manifests. By providing a realistic representation of comorbidity relationships and how patients experience treatment for these conditions, these networks can inform the development of clinical trials, ensuring that populations experiencing comorbidities can benefit from innovative therapeutic approaches. This shift in focus from description to practical information is crucial to unlocking the true potential of comorbidity networks in HF research.

## Data Availability

No datasets were generated or analysed during the current study.

## Glossary of Terms

Network: A collection of nodes (entities) connected by edges (relationships)
Node: In comorbidity networks, a node typically represents a disease or condition
Edge: A connection between nodes, often representing a statistical relationship (e.g., correlation, co-occurrence) between diseases
Degree: The number of connections a node has to other nodes in the network
Betweenness: A measure of a node’s centrality, indicating how often it lies on the shortest path between other nodes
Closeness: A measure of how close a node is to all other nodes in the network
Hub: A node with a high degree of connectivity, often representing a disease with many comorbid relationships
Module: A group of nodes that are more densely connected to each other than to the rest of the network
Cluster: A group of similar nodes, often identified through algorithmic analysis of the network structure
Network motif: Recurring patterns of interconnections in a network
Phenotype: Observable characteristics of an organism resulting from the interaction of its genotype with the environment
Multi-layer network: A network with multiple types of relationships or data integrated into a single structure
Directed network: A network where edges have a direction, indicating a causal or temporal relationship
Weighted network: A network where edges have associated values indicating the strength of relationships
Random walk: A mathematical method used to explore network structures and identify important nodes or relationships
